# Construction of a CXCL12-KDEL Fusion Gene to Inhibit Head and Neck Squamous Cell Carcinoma Metastasis by Intracellular Sequestration of CXCR4

**DOI:** 10.1155/2015/195828

**Published:** 2015-03-19

**Authors:** Wenchao Zhang, Xudong Wang, Kai Yue, Su Liu, Xiaonan Liu

**Affiliations:** Department of Maxillofacial and ENT, National Cancer Clinical Research Center, Tianjin Medical University Cancer Institute & Hospital, Tianjin 300060, China

## Abstract

The CXCL12-CXCR4 biological axis consisting of the chemotactic factor CXCL12 and its specific receptor CXCR4 plays an important role in oral cancer metastasis. High expression of CXCR4 may help oral squamous cancer cells invade local tissues and metastasize to lymph nodes. No obvious association was observed between CXCL12 expression and lymph node metastasis, suggesting that CXCL12 chemotaxis may only be related to CXCR4 expression on the tumor cell membrane. KDEL can be retained by receptors on the surface of the intracellular endoplasmic reticulum (ER) and also be called an ER retention signal sequence. So we adopted the KDEL sequence in this study to generate a CXCL12-KDEL fusion protein in combination with a traceable E-tag label. As such, CXCL12 was retained in the ER. Specific receptor CXCR4 binds to the CXCL12-KDEL, was also retained in the ER, and was thus prevented from reaching the oral squamous cancer cell surface. We reduced the cell surface level of CXCR4 and called the technique “intracellular sequestration.” By this way, we have finished blocking of CXCL12-CXCR4 biological axis and inhibiting lymph node metastasis of oral carcinoma.

## 1. Introduction

Head and neck squamous cell carcinoma (HNSCC) is the most common malignant tumor of the oral cavity and throat, and subsequent neck lymph node metastases have important influence on prognosis [1–4]. The CXCL12-CXCR4 biological axis consisting of the chemotactic factor CXCL12 and its specific receptor CXCR4 plays an important role in cancer metastasis [5–7]. This axis facilitates tumor metastasis in breast cancer, non-small cell lung cancer, rhabdomyosarcoma, and other human malignant tumors, and the blocking of CXCL12-CXCR4 biological axis inhibits metastasis [8–11].

KDEL signal sequence is located in the carboxyl end of structural and functional proteins in the endoplasmic reticulum (ER). It represents a four-peptide sequence: Lys-Asp-Glu-Leu. Relevant receptors for the sequence in the Golgi membrane can recognize KDEL signals and combine with them, and then the combined ER proteins will be carried back to ER. KDEL can be retained by receptors on the surface of the intracellular ER and also be called an endoplasmic reticulum (ER) retention signal sequence.

Here we have made use of “intracellular sequestration” to reduce the cell surface level of CXCR4 by constructing CXCL12-KDEL fusion gene. Specific receptor CXCR4 binds to the CXCL12-KDEL, is also retained in the ER, and is thus prevented from reaching the Tb squamous cancer cell surface.

We aim to analyze the role of the CXCL12-CXCR4 biological axis on HNSCC lymph node metastasis. This will be achieved by constructing and utilizing a CXCL12-KDEL fusion gene expression vector (CXCL12-KDEL-pIRES2-EGFP) to block the CXCL12-CXCR4 biological axis and intracellularly sequester CXCR4, in order to inhibit HNSCC metastasis.

## 2. Materials and Methods

### 2.1. General Data

All the samples were collected from patients admitted to the Tianjin Medical University Cancer Hospital between January 2005 and December 2006. Tissue samples surgically removed from 65 patients with HNSCC and 15 patients with benign lesions were included in this study, as the experimental and control groups, respectively. There were 43 men and 22 women, with an average age of 61 y (range: 19 to 83 y). Patients were staged according to the TNM staging criteria (2012) designed by the Union for International Cancer Control (UICC). In all, there were 26 cases in stages I-II and 39 cases in stages III-IV. Among the 65 patients of squamous cell carcinomas, 35 patients had ipsilateral and/or contralateral neck lymph node metastases, 30 patients had no lymph node metastases, and 2 patients had distant metastases. None of the patients received preoperative chemotherapy and radiotherapy. The carcinoma diagnosis was histopathologically confirmed with complete clinical and pathological data.

### 2.2. Experimental Materials

Competent* Escherichia coli* cells JM109, pMD19T plasmid, and DH5*α* cell were purchased from Takara Shuzo Co., Ltd. (Kyoto, Japan) and Promega (Madison, WI, USA), respectively. PIRES2-EGFP plasmid was prepared in our laboratory. Superscript II reverse transcription kit and PCR products extraction kit were purchased from Qiagen (Hilden, Germany) and Invitrogen Corporation (Maryland, USA), respectively. PCR purification and DNA connection kits were purchased from Takara Shuzo Co., Ltd. (Kyoto, Japan) and Roche Company (USA), respectively. RPMI-1640 culture medium was purchased from Invitrogen Company (USA) and fetal bovine serum (FBS) was purchased from Gibco Company (USA). Human tongue squamous cancer cell line Tb was provided by Shanghai Jiaotong University affiliated Ninth People's Hospital. Goat polyclonal antibody against CXCL12 was purchased from Santa Cruz Company (USA); horseradish peroxidase-labeled second antibody and mouse anti-*β*-actin antibody polyclonal antibody were purchased from Beijing Golden Bridge Biotechnology Company. RIPA lysis buffer was purchased from Millipore Company (USA).

### 2.3. Experimental Methods

#### 2.3.1. Expression and Localization of CXCL12 and CXCR4 in Primary Tumors and Metastatic Lymph Nodes

*(1) Expression and Localization of CXCL12 and CXCR4 in HNSCC and Lymph Nodes by Immunohistochemistry*. CXCR4 and CXCL12 were detected using a rabbit anti-human polyclonal antibody (Boster Company, Wuhan, China) and rabbit anti-human monoclonal antibody (Santa Cruz, USA), respectively. Experiments were performed according to the manufacturer's instructions.

*(2) CXCR4 mRNA Levels in HNSCC Metastasis Group, HNSCC Nonmetastasis Group, and Control Group and CXCL12 mRNA Levels in Metastatic Lymph Nodes and Nonmetastasis Group by RT-PCR*. Total RNA was extracted using Trizol (Invitrogen, Carlsbad, CA, USA), following the manufacturer's protocol. RT reactions were performed in a final volume of 20 *μ*L using M-MLT reverse transcriptase, according to the manufacturer instructions. The resulting cDNA products were stored at −20°C. The primers used in this study were as follows: endogenous control *β*-actin: 5′-CCTGGGCATGGAGTCCTGTG-3′ (forward), 5′-AGGGGCCGGACTCGTCATAC-3′ (reverse); CXCLl2: 5′-GCCATGAACGCCAAGGTC-3′ (forward), 5′-CGAGTGGGTCTAGCGGAAAG-3′ (reverse), 312 bp; CXCR4; 5′-AGCTGTTGGCTGAAAAGGTGGTCTATG-3′ (forward), 5′-GCGCTTCTGGTGGCCCTTGGAGTGTG-3′ (reverse), 254 bp. PCR amplification of CXCR4 was performed under the following conditions: 94°C for 5 min followed by 30 cycles of 94°C for 1 min, 56°C for 1 min, and 72°C for 1 min, with a final extension at 72°C for 10 min. PCR amplification of CXCL12 was performed under the following conditions: 94°C for 5 min, followed by 30 cycles of 94°C for 1 min, 55°C for 1 min, and 72°C for 1 min, with a final extension at 72°C for 10 min. RT-PCR products were detected by agarose gel electrophoresis.

#### 2.3.2. Construction of the CXCL12-KDEL Fusion Gene

*(1) CXCL12-KDEL Fusion Gene Primer Design and Fragments Amplification*. CXCL12 coding gene sequences were retrieved from GenBank to determine the full amplification sequence. Once this was determined, cellular RNA was used as template, and the CXCL12-KDEL fragment was amplified by RT-PCR, resulting in an amplified fragment of 350 bp. The CXCL12-KDEL fusion gene primers used were as follows: 5′-TAGCAGATCTGCCATGGACGCCAAG-3′ (forward) and 5′-TAGCGTCGACTTACAGCTCGTCCTTCTCGCTTCGCGGTTCCAGCGGATCCGGATACGGCACCGGCGCACCCTTGTTTAAAGCTTTCTCCAGGTA-3′ (reverse); they were synthesized by SBS Genetech Co., Ltd. (Beijing, China). The ER retention sequence KDEL and the fusion gene's detecting marker genes sequence (E-tag) were added to reverse primers. The final PCR amplification reaction consisted of the following components: 5 *μ*L of 10x PCR buffer, 4 *μ*L of 4x dNTP (2.5 mM, each), 3 *μ*L of MgC1_2_ (25 mM), l *μ*L each of P1 and P2 primers (both at 200 pmol), l *μ*L of RNA template, 0.5 *μ*L of Taq polymerase (2.5 U/*μ*L), and 34.5 *μ*L of double distilled water. The PCR reaction conditions were as follows: 94°C for 5 min, followed by 30 cycles of 94°C for 1 min, 62°C for 1 min, and 72°C for 1 min. The resulting 350-bp PCR product was separated and analyzed by electrophoresis on a 1% agarose gel, followed by purification and recovery. 

*(2) Amplification and Identification of Recombinant pMD19-T Vector*. The purified CXCL12-KDEL gene was inserted into the pMD19-T vector to obtain a recombinant vector that was subsequently amplified and sequence verified. 

*(3) Construction of CXCL12-KDEL-pIRES2-EGFP Plasmid Eukaryotic Expression Vector*. The amplified products were then subcloned into a pIRES2-EGFP plasmid, transformed into DH5*α* competent cells, and cultured overnight on a Luria broth agar plate containing kanamycin, in a 37°C constant temperature incubator. Single colonies were picked from the plate, following which plasmid DNA was extracted as per the manufacturer's protocol. The resulting DNA was subjected to restriction digestion using two enzymes,* Bgl*II and* Sal*I, and 5 *μ*L of the digested sample was separated by agarose gel electrophoresis. Positive clones identified by restriction enzyme digestion were then sequenced by Takara Company (Dalian, China). The resulting recombinant construct is referred to as CXCL12-KDEL-pIRES2-EGFP.

#### 2.3.3. Recombinant Plasmid Transfection

*(1) Tb Cell Culture and Gene Transfection*. Tb cells were cultured in RPMI 1640 medium and supplemented with 10% FBS, 4 mM L-glutamine, 50 *μ*/mL penicillin, and 50 *μ*g/mL streptomycin (Invitrogen, Carlsbad, CA, USA) at 37°C under 5% CO_2_. Tb cells in logarithmic growth phase were seeded in 6-well plates, with 3 × 10^5^ cells in each well. When the cells were 80% confluent, growth medium was removed, the cells were washed twice with PBS, and 2 mL of RPMI-1640 medium without serum was added back, and the cells were placed in an incubator at 37°C for 40 min. CXCL12-KDEL-pIRES2-EGFP plasmid (5 *μ*L) was combined with Lipofectamine (10 *μ*L) and added to the cell culture medium. The cells were then incubated at 37°C for 6 h, following which the medium was replaced with fresh complete medium and the cells were incubated for an additional 48 h until harvest. 

*(2) Assay for Transfection Efficiency*. Tb3.l cells cultured for 48–72 h after transfection with CXCL12-KDEL-pIRES2-EGFP plasmid were evaluated for transfection efficiency by measuring levels of fluorescent protein expression using a DP70 fluorescence inverted phase contrast microscope. Fluorescent and bright field images were analyzed using the IPP5.1 software (Olympus Company, Japan).

#### 2.3.4. Recombinant Fusion Gene Functional Experiment

*(1) Determination of CXCL12-KDEL Protein Level by Western Blot*. CXCL12-KDEL protein levels at 72 h after transfection were evaluated by measuring levels of the E-tag label using western blot. Cells were divided into six groups: CXCL12-KDEL-pIRES2-EGFP transfection group, empty vector pIRES2-EGFP transfection group, nontransfection group, and culture supernatants from each of the three groups. 

*(2) Analysis of Surface CXCR4 Expression in Transfected Cells*. Transfected cells were harvested and incubated with CXCR4 antibody, following which they were analyzed by flow cytometry. Cells transfected with CXCL12-KDEL-pIRES2-EGFP were compared to cells transfected with the empty vector pIRES2-EGFP. 

*(3) Cell Chemotaxis Assay*. For the chemotaxis assay, cancer cells were added to the upper layer of a chemotaxis chamber and recombinant CXCL12 was added to the bottom layer. Cells were counted and analyzed after incubation at 37°C for 2 h. Cells were divided into three groups: CXCL12-KDEL-pIRES2-EGFP transfection group, empty vector pIRES2-EGFP transfection group, and nontransfection group.

### 2.4. Statistical Analysis

All the statistical analyses were performed using SPSS 16.0 (SPSS, Chicago, IL, USA). IHC results were estimated using *χ* ^2^ test or Fisher's exact test; RT-PCR results were estimated using ANOVA and two-sample* t*-test. A *P* value of <0.05 indicates a significant difference.

## 3. Results

### 3.1. Immunohistochemical Results

**Figure 1:**
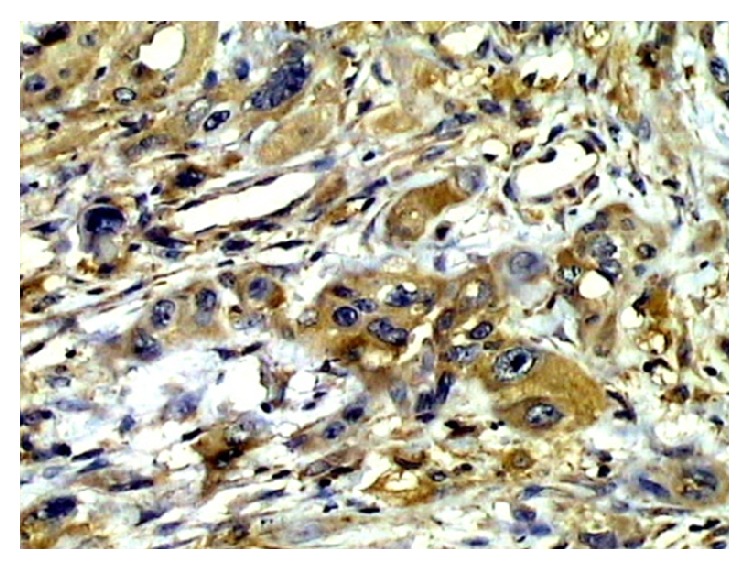
Strong positive expression of CXCR4 in metastatic squamous carcinoma tissues (200x).

**Figure 2:**
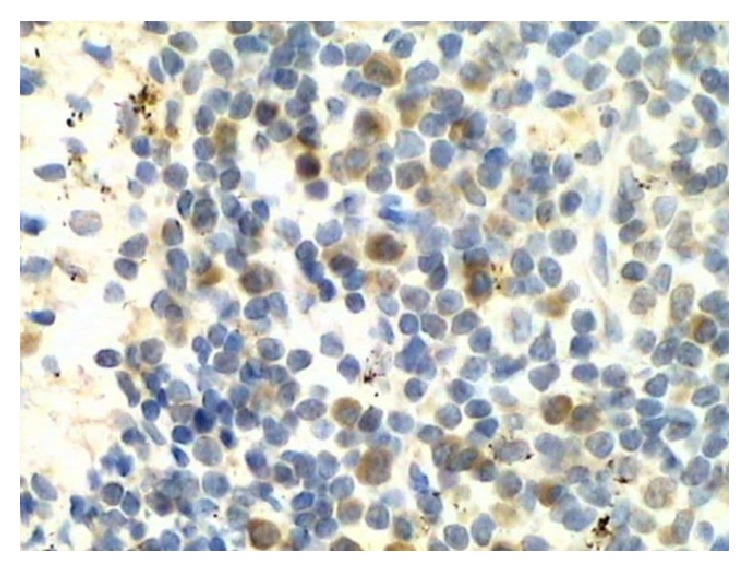
Positive expression of CXCL12 in lymph node tissues (200x).

65 patient's specimens were tested by IHC. CXCR4 was mostly expressed in squamous carcinoma tissues and localized mainly to the cytoplasm and partially to the cell membrane (Figure 1). CXCL12 was expressed in lymph node tissues, primarily in lymphocytes, and localized to the intercellular compartments (Figure 2).

Statistical analysis showed that the positive expression of CXCR4 in stages III-IV was significantly higher than that in stages I-II group (*P* = 0.00). Similarly, it was higher in G3 group than that in G1-G2 group (*P* = 0.00) and higher in metastatic group than that in the nonmetastatic group (*P* = 0.017) (Table 1).

There were no significant differences of CXCL12 positive expression in all compared groups (*P* > 0.05) (Table 1).

### 3.2. RT-PCR

**Figure 3:**
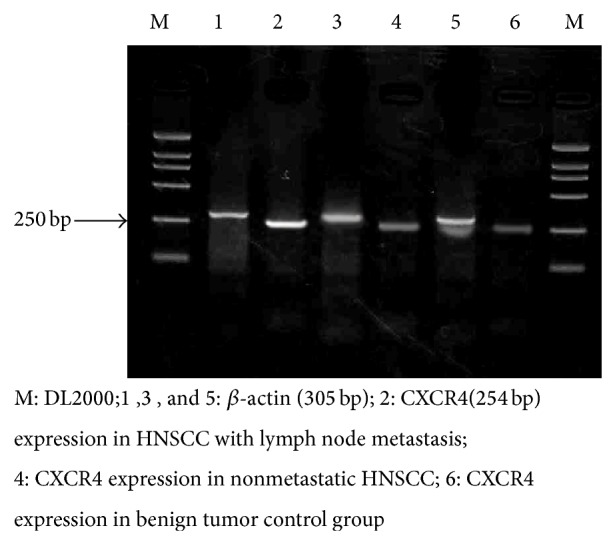
CXCR4 expression in primary head and neck squamous carcinoma (HNSCC) tissues.

Statistical analysis of integral optical density (IOD) showed that CXCR4 expression in metastatic group was higher than that in the nonmetastatic group and control group (*P* < 0.05); CXCR4 expression in the nonmetastatic group was significantly higher than that in the positive control group (*P* < 0.05) (as shown in Figure 3 and Table 2).

**Figure 4:**
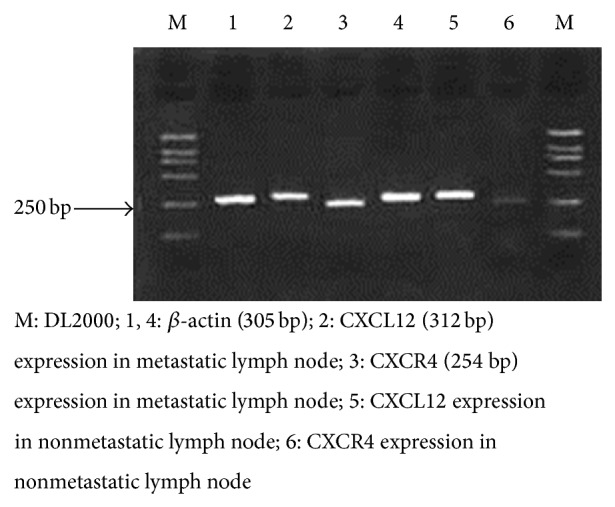
CXCL12 and CXCR4 expression levels in neck lymph nodes.

Statistical analysis showed that the expression of CXCR4 in lymph nodes with metastatic tumor was significantly higher than that of the nonmetastatic lymph nodes (*P* < 0.05), and there was no significant difference of CXCL12 expression in lymph nodes between the two groups (*P* > 0.05) (as shown in Figure 4 and Table 3).

### 3.3. Amplification of CXCL12-KDEL Gene

**Figure 5:**
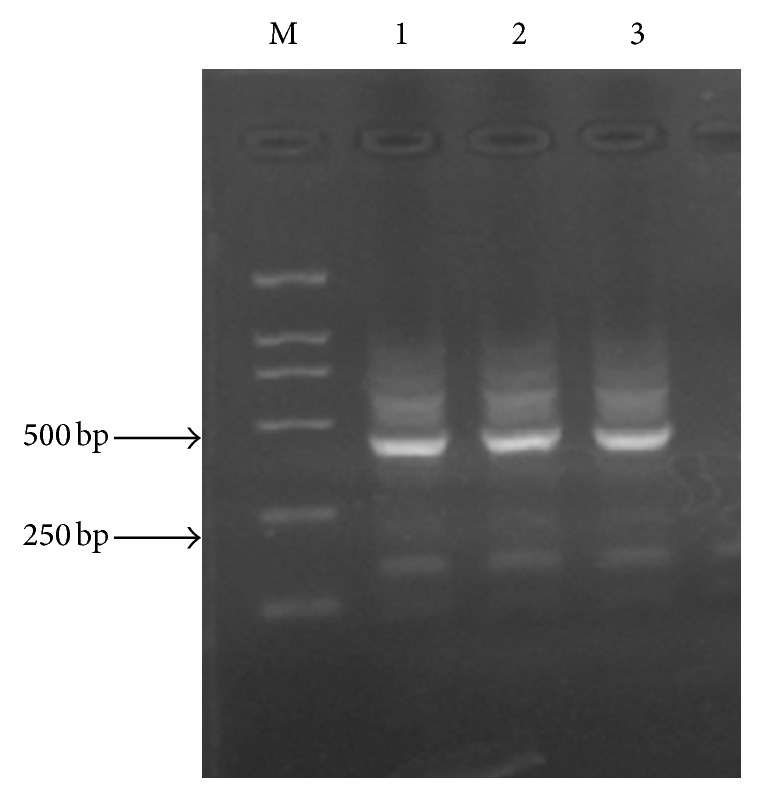
Agarose gel electrophoretogram of CXCL12-KDEL fusion gene PCR products. M: DL2000; 1–3: CXCL12-KDEL (350 bp).

The expected and observed size of the amplified fragment were 350 bp (Figure 5).

### 3.4. Verification of Recombinant Plasmid by Enzyme Digestion and Sequencing

**Figure 6:**
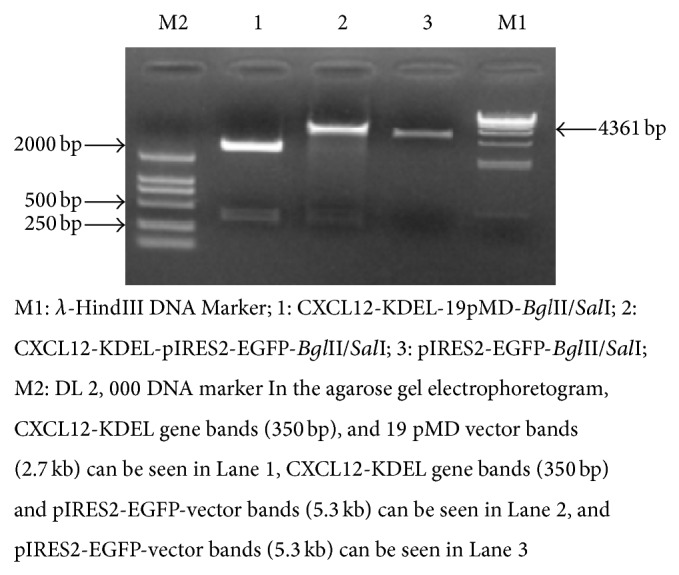
Recombinant 19 pMD and pIRES2-EGFP-vector double digest gel electrophoretogram.

A* Bgl*II/*Sal*I restriction digest of CXCL12-KDEL-pIRES2-EGFP plasmid yields two predicted fragments of 350 bp and 5.3 kb. The length of the inserted product was confirmed to be of the same size as the predicted enzyme digestion product (Figure 6). The CXCL12-KDEL-pIRES2-EGFP plasmid was also sequence verified to be identical to the known gene sequence.

### 3.5. Determination of CXCL12-KDEL-pIRES2-EGFP Plasmid Transfection Efficiency

**Figure 7:**
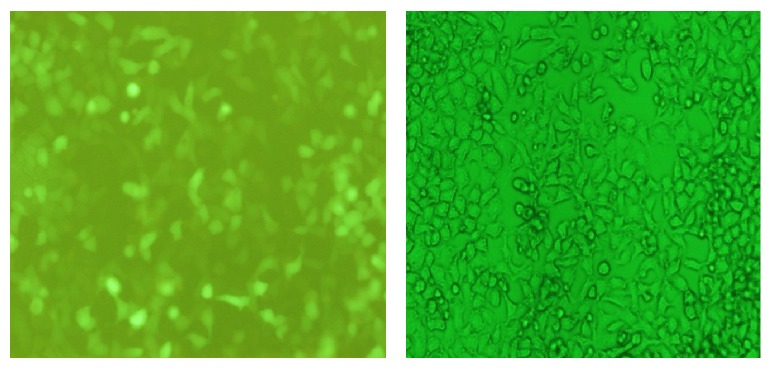
Analysis of Tb cells 72 h after transfection of recombinant CXCL12-KDEL-pIRES2-EGFP plasmid.

We observed that 48 h after recombinant plasmid transfection, 45% of the cells were positive for the expression of green fluorescent protein in Tb cells, and at 72 h after transfection this had increased to 50% (Figure 7).

### 3.6. Determination of CXCL12 Protein Levels by Western Blot

**Figure 8:**
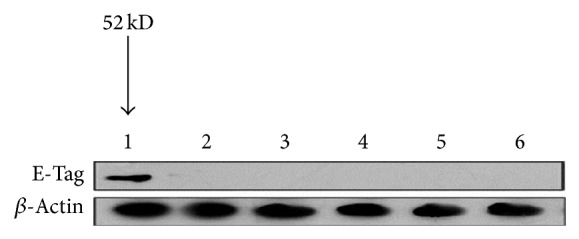
Western blot electrophoretogram of E-tag protein. 1: E-tag protein (52 kd) expression can be seen in Tb cells in the E-tag labeled CXCL12-KDEL transfection group, while no E-tag protein expression was observed in the remaining 5 groups; 2: red vector pIRES2 EGFP transfection group; 3: normal Tb nontransfection cells; 4–6: the culture supernatant of three kinds of cells.

IOD was collected for each sample after transfection with recombinant CXCL12-KDEL-pIRES2-EGFP. Tb cells showed expression of E-tag protein, which is a surrogate label for CXCL12, whereas no protein expression was detected in the cells and the culture supernatant of the cells transfected with an empty plasmid or of those left untransfected (Figure 8).

### 3.7. CXCR4 Cell Surface Expression

**Figure 9:**
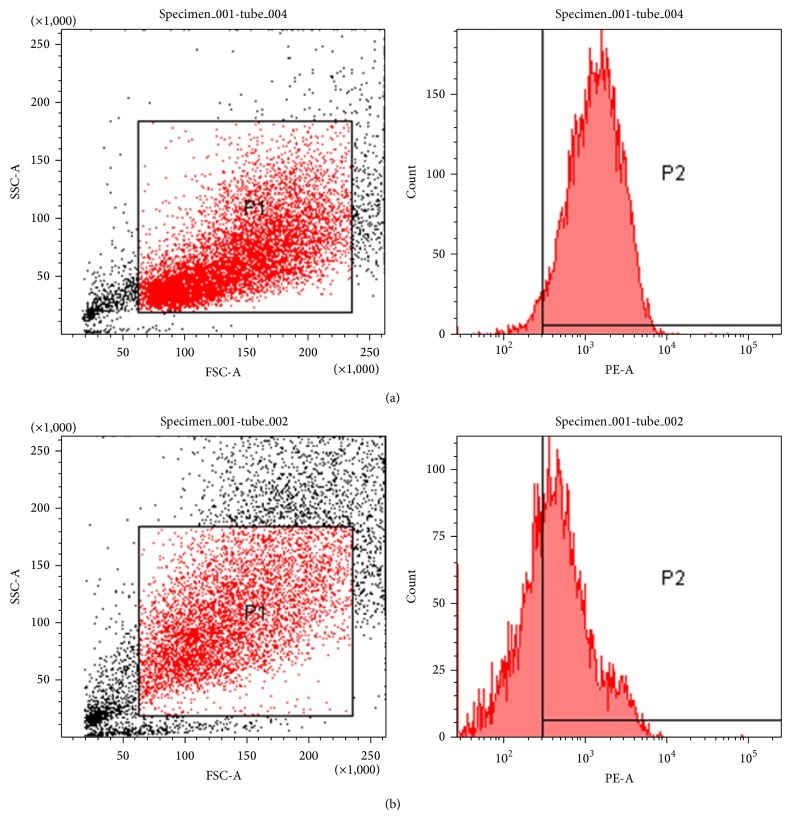
CXCR4 expression on the cell surface after transfection with empty vector (a) or target gene CXCL12-KDEL (b).

Compared with the control group, CXCR4 expression on the cell surface in the experimental group is clearly reduced after transfection (Figure 9).

### 3.8. Chemotaxis Assay

Chemotaxis of cells in the experimental group was significantly less than that in the nontransfection group and the control group (transfected with empty vector) (*F* = 70.14, *P* = 0.00). However, there was no statistically significant difference between the nontransfection group and the empty vector transfection group (Table 4).

## 4. Discussion

Chemokines are a family of small proinflammatory chemoattractant cytokines that bind to leukocyte-expressed seven-transmembrane domain receptors and play a critical role in tumor angiogenesis and metastasis [12]. The chemokine CXCL12, also termed stromal cell derived factor-l (SDF-l), is a member of the CXC chemokine family, of which CXCR4 is a known CXCL12-specific receptor [13, 14]. CXCL12 is a small (8 kDa) chemokine that was originally regarded as an efficacious lymphocyte chemoattractant and was characterized as a modulator of several physiological processes. [15–18]. CXCR4 plays a key role in tumor cell dissemination and metastasis development in the majority of cancers and several types of leukemia [19–23].

Thus, the CXCL12-CXCR4 biological axis formed by CXCL12 and its specific receptor CXCR4 plays an important role in the process of metastasis of cancer cells [24]. The biological axis is related to the invasion, recurrence, and metastasis in many types of cancer including breast cancer [25], non-small cell lung cancer (NSCL) [26], colon cancer [27, 28], oral cavity squamous carcinoma [9], esophageal cancer [29], pancreatic carcinoma [30], renal cell carcinoma [31], and endometrial cancer [32].

In this study, our results show high CXCR4 expression in HNSCC, with higher expression in the metastatic group compared to the nonmetastatic group, which indicates that CXCR4 may play an important role in HNSCC metastasis. No obvious association was observed between CXCL12 expression and lymph node metastasis, suggesting that CXCL12 chemotaxis may only be related to CXCR4 expression on the tumor cell membrane. We speculate that tumor cells with high expression of CXCR4 have strong potential for local invasion, and CXCL12 expressed in lymph nodes has a chemotactic effect on their directional migration.

*In vitro* experiments confirmed that blocking the CXCL12-CXCR4 signal axis activity by different molecular biology methods such as RNAi, small molecule inhibitors in breast cancer cells, could effectively reverse the malignant phenotype of the tumor cells [33–35]. Animal experiments also show that CXCR4 monoclonal antibody can inhibit lymph node metastasis of human breast cancer cells in nude mice. The ER retention signal sequence KDEL represents a four-peptide sequence (Lys-Asp-Glu-Leu), which can be retained by receptors on the surface of the ER [36]. Based on this, we adopted the KDEL sequence in this study to generate a CXCL2-KDEL fusion protein in combination with a traceable E-tag label. As such, CXCL12 was retained in the endoplasmic reticulum allowing it to act as a sink for CXCR4, thus sequestering the latter within the intracellular space. The intracellular sequestration of CXCR4 provides a new strategy for blocking the CXCL12-CXCR4 biological axis. The validity of the CXCL12-KDEL-pIRES2-EGFP plasmid was confirmed by both restriction enzyme digestion and sequencing. At 48 and 72 h after Tb cells were transfected with the recombinant CXCL12-KDEL-pIRES2-EGFP plasmid, we were able to observe fluorescent gene expression from EGFP, confirming successful transfection. Vectors can be used to express EGFP protein alone or obtain stable transfection cell lines. The transfection efficiency can then be analyzed by the intensity of fluorescent protein expression [37]. As determined by western blot of the E-tag label protein, a surrogate marker for CXCL12 protein was successfully expressed following recombinant plasmid transfection, whereas CXCL12 protein expression was not detected in cells transfected with the empty plasmid or in those that were left untransfected.

In this study, we adopted a technique that used CXCL12-KDEL as an intrinsic factor for the intracellular sequestration of CXCR4. We inserted the KDEL sequence downstream of CXCL12 and through functional experiments were able to demonstrate the intracellular capture of CXCR4 surface expression and the blocking of the chemotaxis of cells via the CXCL12-CXCR4 biological axis [38]. This strategy of sequestering CXCR4 using another intracellular component is an example of technology using inactivated biomolecular molecules; other examples are the use of antisense nucleic acid, ribozymes-mediated negative mutation, and gene knockout technologies. These technologies are very similar to the “intracellular antibody” technology, ultimately aimed to form a phenotypic knockout model. In this study, the transfection efficiency met the basic outcomes of the study; however, the specific transfection efficiency needs to be clarified by further screening. Meanwhile, application of this method in* in vitro* experiments can reduce cell surface expression of CXCR4 and the chemotaxis of tumor cells. As such, our results lay a foundation for further screening and animal experiments.

## 5. Conclusions

High expression of CXCR4 may help squamous cancer cells invade local tissues and metastasize to other tissues, and the intracellular sequestration of CXCR4 by transfection of the CXCL12-KDEL fusion gene may inhibit chemotaxis and metastasis in tongue squamous cancer cells.

## Figures and Tables

**Table 1:** The expression of CXCL12/CXCR4 by IHC in different groups of patients.

Clinicopathological parameter	*n*	Positive (%)		*χ* ^2^		*P* value	
		CXCR4	CXCL12	CXCR4	CXCL12	CXCR4	CXCL12
Gender				2.694	0.756	0.101	0.384
Male	43	23 (53.5)	12 (27.9)				
Female	22	12 (54.5)	6 (27.3)				
Age				1.234	0.618	0.267	0.432
<60 ys	31	16 (51.6)	8 (25.8)				
≥60 ys	34	19 (55.9)	10 (29.4)				
Stage				16.44	0.016	**0.000**	0.900
I-II	26	7 (26.9)	7 (26.9)				
III-IV	39	28 (71.8)	11 (28.2)				
Differentiation				27.41	1.025	**0.000**	0.311
G1-G2	47	20 (42.6)	13 (27.7)				
G3	18	15 (83.3)	5 (27.8)				
Metastasis of lymph node				5.704	0.296	**0.017**	0.587
No	30	10 (33.3)	8 (26.7)				
Yes	35	25 (71.4)	10 (28.6)				

**Table 2:** RT-PCR results of CXCR4 expression level in oral squamous cell carcinomas.

Group	*n*	CXCR4 IOD
Normal oral tissues and benign lesions	15	0.406 ± 0.044^∗ ∗∗^
Nonmetastatic SCC	15	0.464 ± 0.068^*^
Lymph node metastatic SCC	15	0.900 ± 0.108^**^

SCC: squamous cell carcinomas and ^∗,∗∗^ *P* < 0.05.

^**^VS ^*^ *P* < 0.05, ^**^VS ^***^ *P* < 0.05, ^*^VS ^***^ *P* < 0.05.

**Table 3:** RT-PCR results of CXCL12 and CXCR4 expression level in neck lymph nodes (IOD).

Group	*n*	CXCL12^*^	CXCR4^**^
Metastatic lymph nodes	7	0.935 ± 0.087	0.947 ± 0.042
Nonmetastatic lymph nodes	7	0.861 ± 0.047	0.396 ± 0.071

^*^ *P* > 0.05, ^**^ *P* < 0.05.

**Table 4:** Chemotactic cells count and chemotactic index in each group ($\bar{x}$  ± s, *n* = 3).

Group	Cell count	CI value
Nontransfection group	825.67 ± 62.80	1
Empty vector (pIRES2-EGFP) transfection group	711.33 ± 49.54	0.86
CXCL12-KDEL transfection group	216.00 ± 84.12	0.26
